# Mean Consistency of Estimators in a Partially Linear Model with AANA Errors

**DOI:** 10.3390/e28070776

**Published:** 2026-07-08

**Authors:** Yu Zhang, Zhiqi Chen

**Affiliations:** 1 School of Mathematics and Statistics, Institute of Big Data Analysis and Applied Mathematics, Hubei University of Education, Wuhan 430205, China; zhangyu@hue.edu.cn; 2 Institute of Scientific Research, Nanjing University of Finance & Economics, Nanjing 210023, China

**Keywords:** AANA, partially linear model, least squares estimators, weighted least squares estimators, *p*-th mean consistency, 62G05, 62G20, 62F12

## Abstract

This paper focuses on a heteroscedastic partially linear regression model in which the errors are asymptotically almost negatively associated (AANA) random variables with a stochastically dominated and zero mean. Under some suitable conditions, the p-th $\left(p>0\right)$ mean consistency of least squares estimators and weighted least squares estimators for the unknown parameter is established, and the p-th $\left(p>0\right)$ mean consistency of the estimators for non-parametric components is also obtained. In addition, the moment convergence rate of the estimators is also investigated. Some results derived in this paper extend and improve the corresponding ones of negatively associated (NA) random errors and independent random errors. Finally, a simulation is carried out to study the numerical performance of the results that we have established.

## 1. Introduction

### 1.1. Partially Linear Regression Model

Consider the following heteroscedastic partially linear regression model:

(1) $$y^{\left(t\right)}\left(x_{in},z_{in}\right)=z_{in}\beta +h\left(x_{in}\right)+\sigma_{in}\varepsilon^{\left(\left.t\right)\right.}\left(x_{in}\right),\;1\leq t\leq r,\;1\leq i\leq n,$$

where $\sigma_{in}^{2}=f\left(u_{in}\right)$, $z_{in}\in R$, $x_{in}\in R^{d}$, $u_{in}\in R^{d}$, and $\left(x_{in},\;z_{in},\;u_{in}\right)$ are known and nonrandom design points, β is an unknown parameter, $f\left(\cdot \right)$ and $h\left(\cdot \right)$ are unknown functions defined on a compact set $M\subseteq R^{d}$, $y^{\left(t\right)}\left(x_{in},\;z_{in}\right)$ denotes the t-th response variables that are observable at points $\left(x_{in},\;z_{in}\right)$, and $\left\{\varepsilon^{\left(t\right)}\left(x_{in}\right),\;1\leq t\leq r,\;1\leq i\leq n\right\}$ denotes asymptotically almost negatively associated random errors with a stochastically dominated and zero mean.

To analyze the relationship between temperature and electricity usage, Engle et al. [1] first introduced the following partially linear regression model:

(2) $$y_{i}=x_{i}^{\prime }\beta +h\left(z_{i}\right)+\varepsilon_{i},\;1\leq i\leq n.$$

Since then, many statisticians pay attention to studying partially linear regression models. Model (2) was further studied by Heckman [2], Speckman [3], Gao [4], Härdle et al. [5], Hu et al. [6], Zeng and Liu [7], and so forth. Some applications of the model were given. Inspired by model (2), a more general partially linear regression model was proposed by Gao et al. [8]:

(3) $$y_{i}=x_{i}\beta +h\left(z_{i}\right)+\sigma_{i}\varepsilon_{i},\;1\leq i\leq n.$$

Gao et al. [8] established the asymptotic normality for the least squares estimators and weighted least squares estimators of β based on the family of non-parametric estimators for $h\left(\cdot \right)$ and $f\left(\cdot \right)$ in model (3). Baek and Liang [9] investigated the strong consistency and asymptotic normality of the estimators in model (3) under negatively associated samples. Zhou et al. [10] derived the moment consistency of the estimators in model (3) with NA errors. Hu [11] proposed a new partially linear regression model,

(4) $$y^{\left(t\right)}\left(x_{in},z_{in}\right)=z_{in}\beta +h\left(x_{in}\right)+\varepsilon^{\left(t\right)}\left(x_{in}\right),\;1\leq t\leq r,\;1\leq i\leq n,$$

and established the strong consistency and moment consistency for the estimators with independent and φ-mixing errors. Li and Yang [12,13] studied the strong consistency and moment consistency of the estimators for β and $h\left(\cdot \right)$ in model (4) with NA errors. Wang et al. [14] and Wu and Wang [15] discussed the moment consistency and strong consistency for least squares estimators and weighted least squares estimators of β and $h\left(\cdot \right)$ with $\tilde{\rho }$-mixing errors. Based on model (4), Zhou et al. [16] considered model (1), obtained the moment consistency of the estimators with negatively associated errors, and noted that model (1) can be used in hydrology, biology, and so on. For more details about regression models, one can refer to [17,18], and so on. Asymptotically almost negatively associated sequences are widely used dependent sequences, which include independent and negatively associated sequences as special cases. Thus, studying the limit properties of the estimators in model (1) for asymptotically almost negatively associated samples has higher theoretical significance and application value. The concept of asymptotically almost negatively associated sequences of random variables was introduced by Chandra and Ghosal [19] as follows.

### 1.2. Concept of AANA Sequences

Now, let us recall two concepts of dependent structures.

**Definition** **1** ([20])**.** *A finite collection of random variables* $X_{1},X_{2},\cdots ,X_{n}$* is said to be negatively associated (NA) if for every pair of disjoint subsets* $A_{1}$* and* $A_{2}$* of* $\left\{1,2,\cdots ,n\right\}$*, *

$$Cov\left\{f\left(X_{i},i\in A_{1}\right),g\left(X_{j},j\in A_{2}\right)\right\}\leq 0,$$

*whenever* f *and* g *are coordinatewise non-decreasing such that this covariance exists. An infinite sequence* $\left\{X_{n},n\geq 1\right\}$ *is NA if every finite subcollection is NA.*

**Definition** **2** ([19])**.** *A sequence* $\left\{X_{n},n\geq 1\right\}$* of random variables is called asymptotically almost negatively associated (AANA) if there exists a non-negative* $q\left(n\right)\to 0$* as* n→∞* such that*

$$Cov\left(f\left(X_{n}\right),g\left(X_{n+1},X_{n+2},\cdots ,X_{n+k}\right)\right)\;\leq q\left(n\right){\left[Var\left(f\left(X_{n}\right)\right)Var\left(g\left(X_{n+1},X_{n+2},\cdots ,X_{n+k}\right)\right)\right]}^{1/2}$$

*for all* n, k≥1 *and for all coordinatewise non-decreasing continuous functions* f *and* g *whenever the variances exist.*

Chandra and Ghasal [19] pointed out that the family of AANA sequences contains NA (in particular, independent) sequences (with $q\left(n\right)\equiv 0,\;n\geq 1$) and some more sequences of random variables. Two examples of AANA sequences that are not NA were constructed by Chandra and Ghosal: $\xi_{n}={\left(1+a_{n}^{2}\right)}^{-1/2}\left(\eta_{n}+a_{n}\eta_{n+1}\right)$(see Chandra and Ghasal [19]) and $\zeta_{n}=\eta_{n}+a_{n}\eta_{n+1}$(see Chandra and Ghasal [20]), where $\eta_{1},\eta_{2},\ldots$ are independent and identically distributed $N\left(0,1\right)$ random variables, and $a_{n}>0$ and $a_{n}\to 0$ as n→∞.

Many applications of AANA sequences have been found. The Kolmogorov-type inequality and Marcinkiewcz–Zygmund-type strong laws of large numbers were derived by Chandra and Ghosal [19]. An [21] studied the complete moment convergence of weighted sums for processes under AANA assumptions. Wang et al. [22] investigated the large deviation and Marcinkiewicz-type strong law of large numbers for AANA sequences. Ko et al. [23] established the Hájeck–Rènyi inequalities for AANA sequences. The complete convergence for AANA sequences was investigated by Shen and Wu [24]. Some Rosenthal-type inequalities for maximum partial sums of AANA sequences were provided by Yuan and An [25]. The laws of large numbers for Ces`aro alpha-integrable random variables under dependence condition AANA or AQSI were studied by Yuan and An [26]. Xi et al. [27] investigated the $L^{p}$ convergence and complete convergence for weighted sums of AANA random variables. Wang et al. [28] obtained the complete convergence and complete moment convergence of weighted sums for an array of row-wise AANA sequences. Zhang et al. [29] established the weak consistency of the M-estimator in a linear regression model with AANA errors. Hu and Zhang [30] obtained the strong consistency of the M-estimator in linear regression model with AANA errors, and so on.

However, we have not found studies on the moment consistency of the estimators for parametric and non-parametric components in model (1) with AANA random errors in the literature. In this paper, we consider the estimation problem for model (1) under the assumption that the errors are AANA sequences of random variables with a stochastically dominated and zero mean. The p-th $\left(p>0\right)$ mean consistency of least squares estimators and weighted least squares estimators for β and $h\left(\cdot \right)$ is derived, respectively, based on some suitable conditions. In addition, the moment convergence rate of the estimators for β and $h\left(\cdot \right)$ is also studied. Some results extend and improve the corresponding ones of Zhou et al. [16] for NA random errors.

The following concept of stochastic domination is used in this work.

**Definition** **3** ([31])**.** *A sequence* $\left\{X_{n},n\geq 1\right\}$* of random variables is said to be stochastically dominated by a random variable* X* if there exists a positive constant* C* such that *

$$P\left(\left|X_{n}\right|>x\right)\leq CP\left(\left|X\right|>x\right)$$

*for all* x≥0 *and* n≥1*.*

The remainder of this paper is organized as follows. The least squares estimators and weighted least squares estimators of β based on the family of non-parametric estimators for $h\left(\cdot \right)$ and some assumptions are introduced in Section 2. We give some preliminary Lemmas in Section 3. The main results are given in Section 4. A simulation is presented in Section 5. We provide the proofs of the main results and the Lemmas in Section 6 and Section 7, respectively. Throughout this paper, let C be a positive constant whose values may vary at different places.

## 2. Estimation and Conditions

Assume that $\left\{y^{\left(t\right)}\left(x_{in},z_{in}\right),z_{in}\in R,x_{in}\in M,u_{in}\in M,1\leq t\leq r,1\leq i\leq n\right\}$ satisfies model (1) and $W_{nj}\left(x\right)=W_{nj}\left(x;x_{1},x_{2},\cdots ,x_{n}\right)$ is a measurable weight function on the compact set M. For simplicity and convenience, model (1) can be written as

(5) $$y_{i}^{\left(t\right)}=z_{i}\beta +h\left(x_{i}\right)+\sigma_{i}\varepsilon_{i}^{\left(t\right)},\;1\leq t\leq r,\;1\leq i\leq n.$$

We denote ${\tilde{z}}_{j}=z_{j}-\sum_{i=1}^{n}W_{ni}\left(x_{j}\right)z_{i}$, ${\tilde{y}}_{j}^{\left(k\right)}=y_{j}^{\left(k\right)}-\frac{1}{r}\sum_{t=1}^{r}\sum_{i=1}^{n}W_{ni}\left(x_{j}\right)y_{i}^{\left(t\right)}$, $\gamma_{i}=1/\sigma_{i}^{2}$, 1≤k≤r, 1≤j≤n, ${\tilde{T}}_{n}^{2}=\sum_{i=1}^{n}{\tilde{z}}_{i}^{2}$, and ${\tilde{U}}_{n}^{2}=\sum_{i=1}^{n}\gamma_{i}{\tilde{z}}_{i}^{2}$.

For model (5), one can obtain from $E\varepsilon_{i}^{\left(t\right)}=0$ that $h\left(x_{i}\right)=E\left(y_{i}^{\left(t\right)}-z_{i}\beta \right)$ for 1≤t≤r 1≤i≤n. Hence, for any given β, a natural non-parametric estimator of $h\left(\cdot \right)$ can be defined by

(6) $$h_{r,n}\left(x,\beta \right)=\frac{1}{r}\sum_{t=1}^{r}\sum_{i=1}^{n}W_{ni}\left(x\right)\left(y_{i}^{\left(t\right)}-z_{i}\beta \right).$$

To obtain the least squares (LS) estimators of β, we seek to minimize

$$\sum_{t=1}^{r}\sum_{i=1}^{n}{\left[y_{i}^{\left(t\right)}-z_{i}\beta -h_{r,n}\left(x_{i},\beta \right)\right]}^{2}.$$

Hence, the least squares (LS) estimators of β can be defined by

(7) $${\hat{\beta }}_{r,n}^{\left(LS\right)}=\arg\underset{\beta }{\min}\sum_{t=1}^{r}\sum_{i=1}^{n}{\left[y_{i}^{\left(t\right)}-z_{i}\beta -h_{r,n}\left(x_{i},\beta \right)\right]}^{2}$$

By (7), we have

(8) $${\hat{\beta }}_{r,n}^{\left(LS\right)}=\frac{1}{r}\sum_{t=1}^{r}\sum_{i=1}^{n}{\tilde{z}}_{i}{\tilde{y}}_{i}^{\left(t\right)}/{\tilde{T}}_{n}^{2}.$$

When the random errors are heteroscedastic, we modify ${\hat{\beta }}_{r,n}^{\left(LS\right)}$ to a weighted least squares (WLS) estimators. The WLS estimators of β can be defined by

(9) $${\hat{\beta }}_{r,n}^{\left(WLS\right)}=\arg\underset{\beta }{\min}\sum_{t=1}^{r}\sum_{i=1}^{n}{\left[\left(y_{i}^{\left(t\right)}-z_{i}\beta -h_{r,n}\left(x_{i},\beta \right)\right)/\sigma_{i}\right]}^{2}$$

By (9), we derive that

(10) $${\hat{\beta }}_{r,n}^{\left(WLS\right)}=\frac{1}{r}\sum_{t=1}^{r}\sum_{i=1}^{n}\gamma_{i}{\tilde{z}}_{i}{\tilde{y}}_{i}^{\left(t\right)}/{\tilde{U}}_{n}^{2}.$$

Corresponding to ${\hat{\beta }}_{r,n}^{\left(LS\right)}$ and ${\hat{\beta }}_{r,n}^{\left(WLS\right)}$, we define the estimator of $h\left(\cdot \right)$, respectively, as

(11) $${\hat{h}}_{r,n}\left(x\right)=\frac{1}{r}\sum_{t=1}^{r}\sum_{i=1}^{n}W_{ni}\left(x\right)\left(y_{i}^{\left(t\right)}-z_{i}{\hat{\beta }}_{r,n}^{\left(LS\right)}\right)$$

and

(12) $${\tilde{h}}_{r,n}\left(x\right)=\frac{1}{r}\sum_{t=1}^{r}\sum_{i=1}^{n}W_{ni}\left(x\right)\left(y_{i}^{\left(t\right)}-z_{i}{\hat{\beta }}_{r,n}^{\left(WLS\right)}\right)$$

To obtain our results, the following conditions are sufficient:

$\left(C_{1}\right)$

(i) $\underset{n\to \infty }{\lim}{\tilde{T}}_{n}^{2}/n=C$;
(ii) $0<s_{0}\leq \underset{u\in M}{\inf}f\left(u\right)\leq \underset{u\in M}{\sup}f\left(u\right)\leq S_{0}<\infty$;
(iii) $f\left(\cdot \right)$ and $h\left(\cdot \right)$ are continuous functions on the compact set M;
(iv) $\left|h\left(x_{1}\right)-h\left(x_{2}\right)\right|\leq K\left\|x_{1}-x_{2}\right\|$ for a constant K and any $x_{1},\;x_{2}\in M$, where $\left\|\cdot \right\|$ stands for the Euclidean norm.

$\left(C_{2}\right)$

(i) $\underset{x\in M}{\sup}\sum_{i=1}^{n}\left|W_{ni}\left(x\right)\right|=O\left(1\right)$;
(ii) $\underset{i\geq 1,x\in M}{\sup}\left|W_{ni}\left(x\right)\right|=O\left(n^{-\alpha }\right)$ for some α>0;
(iii) $\underset{i\geq 1,x\in M}{\sup}\left|W_{ni}\left(x\right)\right|=O\left(n^{-\frac{1}{2}}\right)$.

$\left(C_{3}\right)$

(i) $\underset{x\in M}{\sup}\left|\sum_{i=1}^{n}W_{ni}\left(x\right)-1\right|=o\left(1\right)$;
(ii) $\underset{x\in M}{\sup}\sum_{i=1}^{n}\left|W_{ni}\left(x\right)\right|I\left(\left\|x_{i}-x\right\|>\delta \right)=o\left(1\right)$ for any δ>0.

$\left(C_{4}\right)$

(i) $\underset{x\in M}{\sup}\left|\sum_{i=1}^{n}W_{ni}\left(x\right)-1\right|=o\left(n^{-\frac{1}{4}}\right)$;
(ii) $\underset{x\in M}{\sup}\sum_{i=1}^{n}\left|W_{ni}\left(x\right)\right|I\left(\left\|x_{i}-x\right\|>an^{-\frac{1}{4}}\right)=o\left(n^{-\frac{1}{4}}\right)$ for some a>0.

$\left(C_{5}\right)$

$\underset{x\in M}{\sup}\left|\sum_{i=1}^{n}W_{ni}\left(x\right)z_{i}\right|=O\left(1\right)$.

**Remark** **1.** *Conditions *$\left(C_{1}\right)$* are imposed by Härdle et al.* [5]*, Gao et al.* [8]*, Baek and Liang* [9]*, and Zhou et al.* [16]*. Conditions* $\left(C_{2}\right)$ *,* $\left(C_{3}\right)$*,* *and* $\left(C_{5}\right)$* are used by Baek and Liang* [9] *and Zhou et al.* [16]*. Conditions* $\left(C_{4}\right)$* are used by Zhou et al.* [10] *and Zhou et al.* [16]*. Therefore, the above conditions are very mild.*

**Remark** **2.** *From* $\left(C_{1}\right)$ *(i) (ii)* *, we can obtain that*

(13) $${\tilde{T}}_{n}^{-2}\sum_{i=1}^{n}\left|{\tilde{z}}_{i}\right|\leq C$$

*and*

(14) $${\tilde{U}}_{n}^{-2}\sum_{i=1}^{n}\left|\gamma_{i}{\tilde{z}}_{i}\right|\leq C.$$

## 3. Preliminary Lemmas

In this section, we will present some lemmas that will be used to prove the main results of the paper.

**Lemma** **1** (Yuan and An [25])**.** *If* $\left\{X_{i},i\geq 1\right\}$* is an AANA sequence with mixing coefficients* $\left\{q\left(i\right),i\geq 1\right\}$*, then* $\left\{f_{i}\left(X_{i}\right),i\geq 1\right\}$* is still an AANA sequence with mixing coefficients* $\left\{q\left(i\right),i\geq 1\right\}$*, where* $f_{1},f_{2},\cdots$* are non-decreasing or non-increasing functions.*

**Lemma** **2** (Yuan and An [25])**.** *Let* $\left\{X_{i},i\geq 1\right\}$* be an AANA sequence of zero mean random variables with mixing coefficients* $\left\{q\left(i\right),i\geq 1\right\}$*. Then, there exists a positive constant* $C_{p}$* depending only on* p* such that*

$$E\left(\underset{1\leq i\leq n}{\max}{\left|S_{i}\right|}^{p}\right)\leq C_{p}\left\{\sum_{i=1}^{n}E{\left|X_{i}\right|}^{p}+{\left(\sum_{i=1}^{n-1}q^{2-p/2}\left(i\right){\left\|X_{i}\right\|}_{p}\right)}^{p}\right\},$$

*for all* n≥1 *and* 1<p≤2*, where* $S_{i}=\sum_{j=1}^{i}X_{j}$ *and* ${\left\|X_{i}\right\|}_{p}={\left(E{\left|X_{i}\right|}^{p}\right)}^{1/p}$*, such that*

(15) $$E\left(\underset{1\leq i\leq n}{\max}{\left|S_{i}\right|}^{p}\right)\leq C_{p}\left\{\left[1+C{\left(\sum_{i=1}^{n-1}q^{\tilde{p}}\left(i\right)\right)}^{p-1}\right]\sum_{i=1}^{n}E{\left|X_{i}\right|}^{p}+{\left(\sum_{i=1}^{n}EX_{i}^{2}\right)}^{p/2}\right\}$$

*for all* n≥1*,* $2^{k}<p\leq 2^{k+1}$*, and* $\tilde{p}=\left(1/2^{i-1}-2/p\right)p/\left(p-1\right)$*, where integer* k≥1*.*

*In particular, if* $\sum_{i=1}^{\infty }q^{2}\left(i\right)<\infty$ *, then *

(16) $$E\left(\underset{1\leq i\leq n}{\max}{\left|S_{i}\right|}^{p}\right)\leq C_{p}\sum_{i=1}^{n}E{\left|X_{i}\right|}^{p}$$

*for all* n≥1 *and* 1<p≤2 *.*

**Lemma** **3** ([31])**.** *Let* $\left\{X_{n},n\geq 1\right\}$* be a sequence of random variables that is stochastically dominated by a random variable* X*. For any* a>0* and* β>0*, the following two statements hold:*

$$E{\left|X_{n}\right|}^{\beta }I\left(\left|X_{n}\right|\leq a\right)\leq C_{1}\left[E{\left|X\right|}^{\beta }I\left(\left|X\right|\leq a\right)+a^{\beta }P\left(\left|X\right|>a\right)\right],\;\;E{\left|X_{n}\right|}^{\beta }I\left(\left|X_{n}\right|>a\right)\leq C_{2}E{\left|X\right|}^{\beta }I\left(\left|X\right|>a\right),$$

*where* $C_{1}$ *and* $C_{2}$ *are positive constants. Thus,*

$$E{\left|X_{n}\right|}^{\beta }\leq CE{\left|X\right|}^{\beta }$$

*where* C *is a positive constant.*

**Lemma** **4.** *Let *p>1*, *α>0*, and *$\left\{X_{i}^{\left(t\right)},1\leq t\leq r,1\leq i\leq n\right\}$* be an AANA sequence of zero mean random variables with mixing coefficients *$\left\{q^{\left(t\right)}\left(i\right)=q\left(\left(t-1\right)n+i\right),1\leq t\leq r,1\leq i\leq n\right\}$*, which is stochastically dominated by a random variable *X* with *$E{\left|X\right|}^{p}<\infty$*. Assume that there exist some *u>p* and *u≥2* such that *

(17) $$\left\{\begin{array}{l} \sum_{t=1}^{\infty }\sum_{i=1}^{\infty }{\left(q^{\left(t\right)}\left(i\right)\right)}^{2}<\infty ,\;\mathrm{when}\;u=2;\\ \sum_{t=1}^{\infty }\sum_{i=1}^{\infty }{\left(q^{\left(t\right)}\left(i\right)\right)}^{\tilde{u}}<\infty ,\;\mathrm{when}\;2^{k}<u\leq 2^{k+1},\;\tilde{u}=\left(1/2^{k-1}-2/u\right)u/\left(u-1\right),\;k\geq 1. \end{array}\right.$$

*Let *$\left\{c_{ni}\left(\cdot \right),\;1\leq i\leq n,\;n\geq 1\right\}$* be a function array defined on compact set *M.

(i) If
  (18) $$\underset{v\in M}{\sup}\sum_{i=1}^{n}\left|c_{ni}\left(v\right)\right|=O\left(1\right)$$
  and
  (19) $$\underset{i\geq 1,v\in M}{\sup}\left|c_{ni}\left(v\right)\right|=O\left(n^{-\alpha }\right),$$
  then
  (20) $$\underset{\min\left(r,n\right)\to \infty }{\lim}\underset{v\in M}{\sup}E{\left|\frac{1}{r}\sum_{t=1}^{r}\sum_{i=1}^{n}c_{ni}\left(v\right)\varepsilon_{i}^{\left(t\right)}\right|}^{p}=0.$$
(ii) If $\underset{v\in M}{\sup}\sum_{i=1}^{n}\left|c_{ni}\left(v\right)\right|=O\left(1\right)$ and $\underset{i\geq 1,v\in M}{\sup}\left|c_{ni}\left(v\right)\right|=O\left(n^{-\frac{1}{2}}\right)$, then
  (21) $$\underset{\min\left(r,n\right)\to \infty }{\lim}\underset{v\in M}{\sup}E{\left|\frac{n^{\frac{1}{4}}}{r}\sum_{t=1}^{r}\sum_{i=1}^{n}c_{ni}\left(v\right)\varepsilon_{i}^{\left(t\right)}\right|}^{p}=0.$$

**Remark** **3.** *The proof of Lemma 4 is in Section 7.*

## 4. Main Results

**Theorem** **1.** *In model (5), let* p>1 *and* $\left\{\varepsilon_{i}^{\left(t\right)},1\leq t\leq r,1\leq i\leq n\right\}$ *be an AANA sequence of zero mean random variables with mixing coefficients* $\left\{q^{\left(t\right)}\left(i\right)=q\left(\left(t-1\right)n+i\right),1\leq t\leq r,1\leq i\leq n\right\}$ *, which is stochastically dominated by a random variable* ε *with* $E{\left|\varepsilon \right|}^{p}<\infty$ *. Suppose that conditions* $\left(C_{1}\right)$ *(i) (ii) (iii),* $\left(C_{2}\right)$ *(i) (ii), and* $\left(C_{3}\right)$ *hold. If (17) is satisfied for some* u>p *and* u≥2 *, then *

(22) $$\underset{\min\left(r,n\right)\to \infty }{\lim}E{\left|{\hat{\beta }}_{r,n}^{\left(LS\right)}-\beta \right|}^{p}=0$$

*and*

(23) $$\underset{\min\left(r,n\right)\to \infty }{\lim}E{\left|{\hat{\beta }}_{r,n}^{\left(WLS\right)}-\beta \right|}^{p}=0.$$

*Assume further that* $\left(C_{5}\right)$ *holds. Then,*

(24) $$\underset{\min\left(r,n\right)\to \infty }{\lim}\underset{x\in M}{\sup}E{\left|{\hat{h}}_{r,n}\left(x\right)-h\left(x\right)\right|}^{p}=0$$

*and*

(25) $$\underset{\min\left(r,n\right)\to \infty }{\lim}\underset{x\in M}{\sup}E{\left|{\tilde{h}}_{r,n}\left(x\right)-h\left(x\right)\right|}^{p}=0.$$

**Remark** **4.** *Since NA sequences are special AANA sequences with *$q\left(i\right)\equiv 0$* (see Chandra and Ghosal* [19]*), by comparing Theorem 2.1 of Zhou et al.* [16] *with Theorem 1, we have the following generalizations or improvements:*

(i) The NA random errors of Theorem 2.1 by Zhou et al. [16] are extended to AANA random errors;
(ii) p>2 is extended to p>1;
(iii) α=1/2 is extended to α>0.

**Theorem** **2.** *In model (5), let *p>1* and *$\left\{\varepsilon_{i}^{\left(t\right)},1\leq t\leq r,1\leq i\leq n\right\}$* be an AANA sequence of zero mean random variables with mixing coefficients* $\left\{q^{\left(t\right)}\left(i\right)=q\left(\left(t-1\right)n+i\right),1\leq t\leq r,1\leq i\leq n\right\}$*, which is stochastically dominated by a random variable *ε*. Suppose that conditions *$\left(C_{1}\right)$*, *$\left(C_{2}\right)$* (i) (iii), and *$\left(C_{4}\right)$* hold. If *$E{\left|\varepsilon \right|}^{m}<\infty$* for some *m>2* and (17) is satisfied for some *u>p* and *u≥2*, then *

(26) $$\underset{\min\left(r,n\right)\to \infty }{\lim}E{\left|n^{\frac{1}{4}}\left({\hat{\beta }}_{r,n}^{\left(LS\right)}-\beta \right)\right|}^{p}=0.$$

*Assume further that *$\left(C_{5}\right)$* holds. Then,*

(27) $$\underset{\min\left(r,n\right)\to \infty }{\lim}\underset{x\in M}{\sup}E{\left|n^{\frac{1}{4}}\left({\hat{h}}_{r,n}\left(x\right)-h\left(x\right)\right)\right|}^{p}=0$$

*and*

(28) $$\underset{\min\left(r,n\right)\to \infty }{\lim}\underset{x\in M}{\sup}E{\left|n^{\frac{1}{4}}\left({\tilde{h}}_{r,n}\left(x\right)-h\left(x\right)\right)\right|}^{p}=0.$$

## 5. Numerical Simulation

In this section, we will investigate the numerical performance of the moment consistency for the least squares estimators of β and $h\left(x\right)$ with AANA random errors with a simulation example.

We will simulate a partially linear model

(29) $$y_{i}^{\left(t\right)}=z_{i}\beta +h\left(x_{i}\right)+\sigma_{i}\varepsilon_{i}^{\left(t\right)},\;1\leq t\leq 2n/3,1\leq i\leq n.$$

where β=3.5, $g\left(t\right)=\cos\left(\pi t\right)$, $z_{i}={\left(-1\right)}^{i}\cdot \frac{i}{n}$, $\sigma_{i}=1$, 1≤i≤n, and the random errors are given by

$$\varepsilon_{i}^{\left(t\right)}={\left(1+a_{i}^{2}\right)}^{-1/2}\left(\eta_{i}^{\left(t\right)}+a_{i}\eta_{i+1}^{\left(t\right)}\right),$$

where $\left\{\eta_{i}^{\left(t\right)},1\leq t\leq r,1\leq i\leq n\right\}$ are independent and identically distributed $N\left(0,1\right)$ random variables and $a_{i}=1/i^{2}$. $\left\{\varepsilon_{i}^{\left(t\right)},1\leq t\leq ,1\leq i\leq n\right\}$ has been proved to be an AANA sequence but not an NA sequence (see Chandra and Ghosal [19]).

In particular, we take the weight function $W_{ni}\left(\cdot \right)$ as the following nearest neighbor weight function (see Hu [32]). Without loss of generality, let $M=\left[0,1\right]$ and $x_{i}=\frac{i}{n}$ $\left(1\leq i\leq n\right)$. For each x∈M, we rewrite

$$\left|x_{1}-x\right|,\left|x_{2}-x\right|,\cdots ,\left|x_{n}-x\right|$$

as follows:

$$\left|x_{R_{1}(x)}-x\right|\leq \left|x_{R_{2}\left(x\right)}-x\right|\leq ,\cdots ,\leq \left|x_{R_{n}\left(x\right)}-x\right|.$$

Take $k_{n}=\left[n^{2/3}\right]$ and define the nearest neighbor weight function

$$W_{ni}\left(x\right)=\left\{\begin{array}{ll} \frac{1}{k_{n}}, & \;\mathrm{if}\;\left|x_{i}-x\right|\leq \left|x_{R_{k_{n}}}-x\right|,\\ 0, & \;\mathrm{otherwise}. \end{array}\right.$$

**Figure 1:**
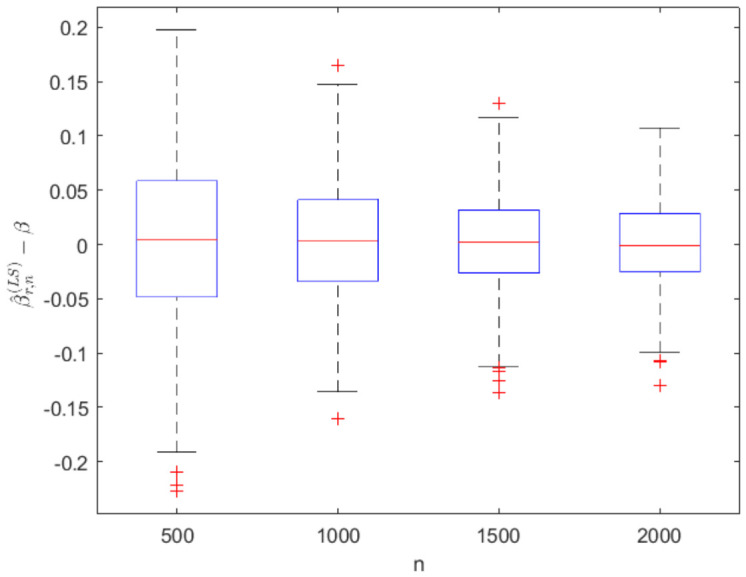
Boxplots of ${\hat{\beta }}_{r,n}^{\left(LS\right)}-\beta$ with β=3.5 and x=0.3.

**Figure 2:**
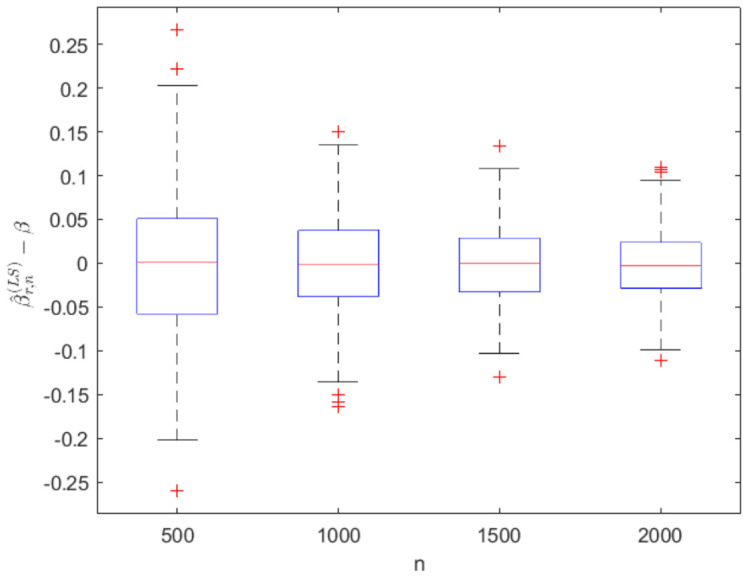
Boxplots of ${\hat{\beta }}_{r,n}^{\left(LS\right)}-\beta$ with β=3.5 and x=0.6.

**Figure 3:**
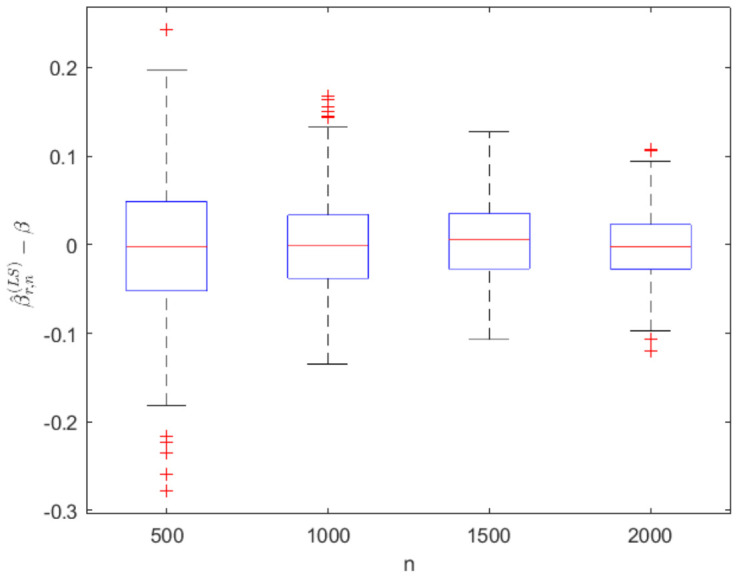
Boxplots of ${\hat{\beta }}_{r,n}^{\left(LS\right)}-\beta$ with β=3.5 and x=0.9.

**Figure 4:**
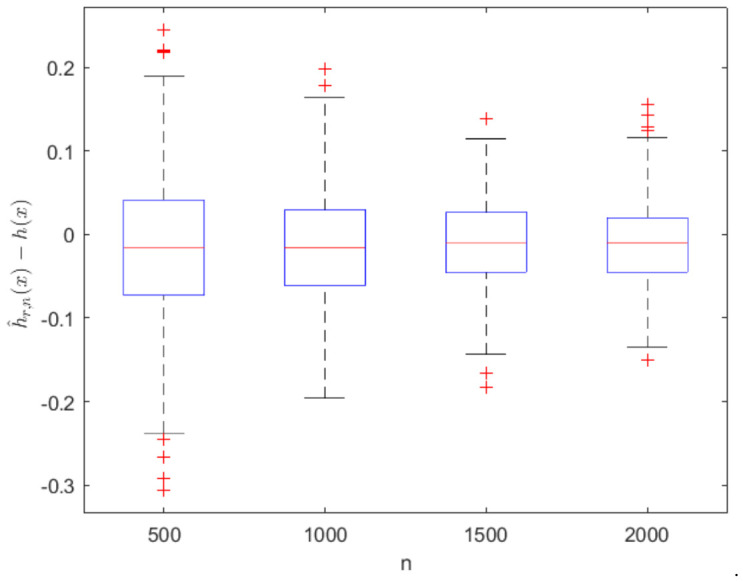
Boxplots of ${\hat{h}}_{r,n}\left(x\right)-h\left(x\right)$ with β=3.5 and x=0.3.

**Figure 5:**
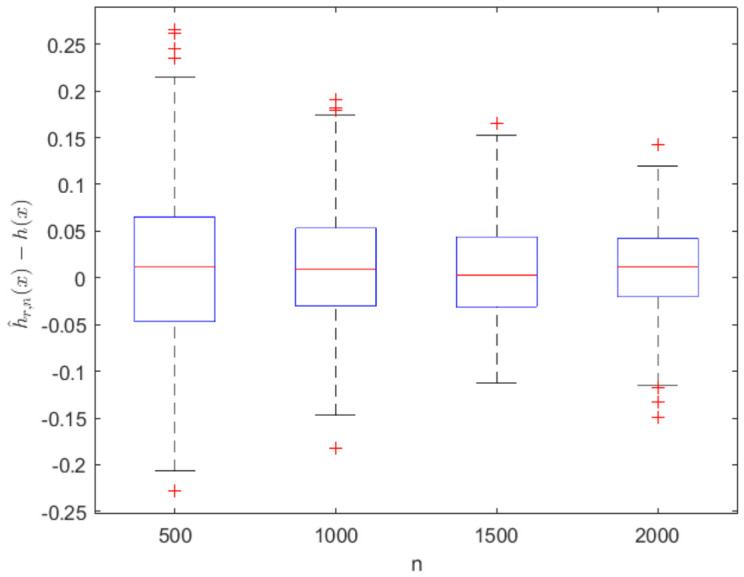
Boxplots of ${\hat{h}}_{r,n}\left(x\right)-h\left(x\right)$ with β=3.5 and x=0.6.

**Figure 6:**
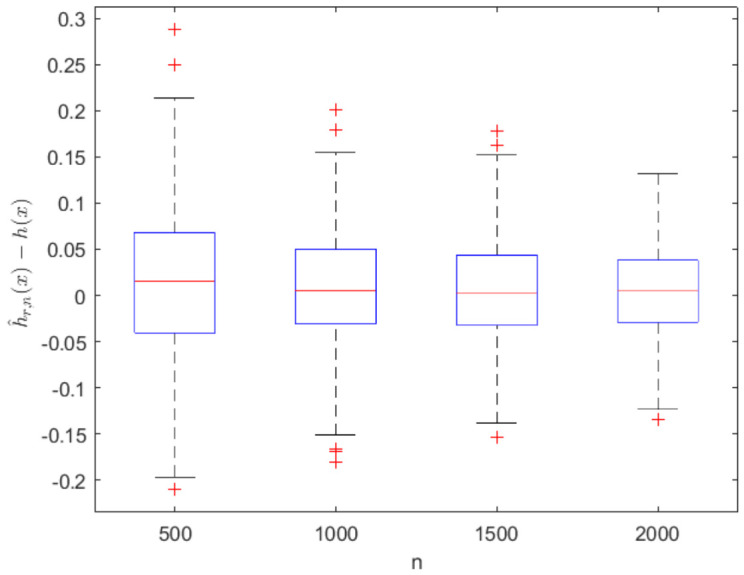
Boxplots of ${\hat{h}}_{r,n}\left(x\right)-h\left(x\right)$ with β=3.5 and x=0.9.

The sample sizes are taken as n=500, 1000, 1500, and 2000, and the points x are taken as x=0.3, 0.6, and 0.9, respectively. We compute ${\hat{\beta }}_{r,n}^{\left(LS\right)}-\beta$ and ${\hat{h}}_{r,n}(x)-h\left(x\right)$ 1000 times, respectively. Their boxplots are provided in Figure 1, Figure 2, Figure 3, Figure 4, Figure 5 and Figure 6 and their mean squared errors (MSEs) are presented in Table 1 and Table 2.

It can be seen from Figure 1 to Figure 6 that regardless of the values of x, ${\hat{\beta }}_{r,n}^{\left(LS\right)}-\beta$ and ${\hat{h}}_{r,n}(x)-h\left(x\right)$ fluctuate to zero line and the ranges of ${\hat{\beta }}_{r,n}^{\left(LS\right)}-\beta$ and ${\hat{h}}_{r,n}(x)-h\left(x\right)$ decrease as the sample size n increases. From Table 1 and Table 2, one can see that regardless of the values of x, the MSEs decrease gradually as the sample size n increases. Thus, the estimators get closer and closer to their real values as the sample size n increases. The simulation results show the mean consistency of least squares estimators ${\hat{\beta }}_{r,n}^{\left(LS\right)}$ and ${\hat{h}}_{r,n}(x)$ in model (1) with AANA random errors. Moreover, consistency is the basic standard that all estimators should meet, and it is the necessary condition to measure whether an estimator is feasible. AANA sequences are widely used dependent sequences that include independent and NA sequences as special cases. Therefore, to study the consistency of the estimators in regression models with AANA errors is of considerable significance.

## 6. Proofs of Theorems

By (5), (8) and (10), we derive that

(30) $${\hat{\beta }}_{r,n}^{\left(LS\right)}-\beta ={\tilde{T}}_{n}^{-2}\left(\frac{1}{r}\sum_{t=1}^{r}\sum_{i=1}^{n}{\tilde{z}}_{i}{\tilde{e}}_{i}^{\left(t\right)}+\sum_{i=1}^{n}{\tilde{z}}_{i}\overset{⌣}{h}\left(x_{i}\right)\right)$$

and

(31) $${\hat{\beta }}_{r,n}^{\left(WLS\right)}-\beta ={\tilde{U}}_{n}^{-2}\left(\frac{1}{r}\sum_{t=1}^{r}\sum_{i=1}^{n}\gamma_{i}{\tilde{z}}_{i}{\tilde{e}}_{i}^{\left(t\right)}+\sum_{i=1}^{n}\gamma_{i}{\tilde{z}}_{i}\overset{⌣}{h}\left(x_{i}\right)\right),$$

where ${\tilde{e}}_{j}^{\left(k\right)}=e_{j}^{\left(k\right)}-\frac{1}{r}\sum_{t=1}^{r}\sum_{i=1}^{n}W_{ni}\left(x_{j}\right)e_{i}^{\left(t\right)}$, $\overset{⌣}{h}\left(x\right)=h\left(x\right)-\sum_{j=1}^{n}W_{nj}\left(x\right)h\left(x_{j}\right)$, and $e_{i}^{\left(t\right)}=\sigma_{i}\varepsilon_{i}^{\left(t\right)}$, 1≤k≤r, 1≤j≤n.

**Proof** **of** **Theorem** **1.** We first prove (23). By (31), we can get that

$$\begin{array}{cc} {\hat{\beta }}_{r,n}^{\left(WLS\right)}-\beta & ={\tilde{U}}_{n}^{-2}\left[\frac{1}{r}\sum_{t=1}^{r}\sum_{i=1}^{n}\gamma_{i}{\tilde{z}}_{i}\sigma_{i}\varepsilon_{i}^{\left(t\right)}-\sum_{j=1}^{n}\gamma_{j}{\tilde{z}}_{j}\left(\frac{1}{r}\sum_{t=1}^{r}\sum_{i=1}^{n}W_{ni}\left(x_{j}\right)\sigma_{i}\varepsilon_{i}^{\left(\left.t\right)\right.}\right)+\sum_{i=1}^{n}\gamma_{i}{\tilde{z}}_{i}\overset{⌣}{h}(x_{i})\right]\\ & ≜I_{r,n}^{\left(1\right)}-I_{r,n}^{\left(2\right)}+I_{r,n}^{\left(3\right)}. \end{array}$$

Hence, it follows by the $C_{p}$-inequality that

(32) $$E{\left|{\hat{\beta }}_{r,n}^{\left(WLS\right)}-\beta \right|}^{p}\leq 3^{p-1}\left(E{\left|I_{r,n}^{\left(1\right)}\right|}^{p}+E{\left|I_{r,n}^{\left(2\right)}\right|}^{p}+E{\left|I_{r,n}^{\left(3\right)}\right|}^{p}\right).$$

Observe that $I_{r,n}^{\left(1\right)}=\frac{1}{r}\sum_{t=1}^{r}\sum_{i=1}^{n}\left({\tilde{U}}_{n}^{-2}\gamma_{i}\sigma_{i}{\tilde{z}}_{i}\right)\varepsilon_{i}^{\left(t\right)}≜\frac{1}{r}\sum_{t=1}^{r}\sum_{i=1}^{n}a_{ni}\varepsilon_{i}^{\left(t\right)}$. Hence, it follows from $\left(C_{1}\right)$ (i) (ii) and (14) that

(33) $$\underset{1\leq i\leq n}{\max}\left|a_{ni}\right|\leq C\underset{1\leq i\leq n}{\max}\left|\gamma_{i}{\tilde{z}}_{i}\right|{\tilde{U}}_{n}^{-1}\cdot {\tilde{U}}_{n}^{-1}=O\left(n^{-1/2}\right)$$

and

(34) $$\sum_{i=1}^{n}\left|a_{ni}\right|\leq C\sum_{i=1}^{n}\left|\gamma_{i}{\tilde{z}}_{i}\right|{\tilde{U}}_{n}^{-2}=O\left(1\right).$$

Thus, by (i) of Lemma 4, we have

(35) $$\underset{\min\left(r,n\right)\to \infty }{\lim}E{\left|I_{r,n}^{\left(1\right)}\right|}^{p}=0.$$

Note that $I_{r,n}^{\left(2\right)}=\frac{1}{r}\sum_{t=1}^{r}\sum_{i=1}^{n}\left(\sum_{j=1}^{n}{\tilde{U}}_{n}^{-2}\gamma_{i}\sigma_{i}{\tilde{z}}_{i}W_{ni}\left(x_{j}\right)\right)\varepsilon_{i}^{\left(t\right)}≜\frac{1}{r}\sum_{t=1}^{r}\sum_{i=1}^{n}a_{ni}^{\prime }\varepsilon_{i}^{\left(t\right)}$. Hence, it follows from $\left(C_{1}\right)$ (i), $\left(C_{2}\right)$ (ii) and (14) that

(36) $$\underset{1\leq i\leq n}{\max}\left|a_{ni}^{\prime }\right|\leq C\underset{i\geq 1,x\in M}{\sup}\left|W_{ni}\left(x\right)\right|\cdot \sum_{i=1}^{n}\left|\gamma_{i}{\tilde{z}}_{i}\right|{\tilde{U}}_{n}^{-2}=O\left(n^{-\alpha }\right)$$

and

(37) $$\sum_{i=1}^{n}\left|a_{ni}^{\prime }\right|\leq C\underset{x\in M}{\sup}\sum_{i=1}^{n}\left|W_{ni}\left(x\right)\right|\cdot \sum_{i=1}^{n}\left|\gamma_{i}{\tilde{z}}_{i}\right|{\tilde{U}}^{-2}=O\left(1\right).$$

Thus, by (i) of Lemma 4, one can get that

(38) $$\underset{\min\left(r,n\right)\to \infty }{\lim}E{\left|I_{r,n}^{\left(2\right)}\right|}^{p}=0.$$

By (14), we derive that

(39) $$\left|I_{r,n}^{\left(3\right)}\right|\leq \underset{x\in M}{\sup}\left|\overset{⌣}{h}\left(x\right)\right|\sum_{i=1}^{n}\left|\gamma_{i}z_{i}\right|/{\tilde{U}}_{n}^{2}\leq C\underset{x\in M}{\sup}\left|\overset{⌣}{h}\left(x\right)\right|.$$

By $\left(C_{1}\right)$ (iii), $\left(C_{2}\right)$ (i), and $\left(C_{3}\right)$, we obtain that

(40) $$\begin{array}{cc} \underset{x\in M}{\sup}\left|\overset{⌣}{h}\left(x\right)\right| & \leq \underset{x\in M}{\sup}\left|\sum_{j=1}^{n}W_{nj}\left(x_{i}\right)-1\right|\left|h\left(x\right)\right|+\underset{x\in M}{\sup}\sum_{j=1}^{n}\left|W_{nj}\left(x\right)\right|\left|h\left(x\right)-h\left(x_{j}\right)\right|\\ & \leq \underset{x\in M}{\sup}\left|\sum_{j=1}^{n}W_{nj}\left(x\right)-1\right|\left|h\left(x\right)\right|\\ & \;+\underset{x\in M}{\sup}\sum_{j=1}^{n}\left|W_{nj}\left(x\right)\right|\left|h\left(x\right)-h\left(x_{j}\right)\right|I\left(\left\|x-x_{j}\right\|>\delta \right)\\ & \;+\underset{x\in M}{\sup}\sum_{j=1}^{n}\left|W_{nj}\left(x\right)\right|\left|h\left(x\right)-h\left(x_{j}\right)\right|I\left(\left\|x-x_{j}\right\|\leq \delta \right)=o\left(1\right) \end{array}$$

Thus, by (39) and (40), we have

(41) $$\underset{\min\left(r,n\right)\to \infty }{\lim}E{\left|I_{r,n}^{\left(3\right)}\right|}^{p}=0.$$

Therefore, (23) follows from (32), (35), (38) and (41).

The proof of (22) is similar to that of (23), so we omit the details here.

Next, we will prove (25). In view of (12), we have

$$\begin{array}{cc} {\tilde{h}}_{r,n}\left(x\right)-h\left(x\right) & =\frac{1}{r}\sum_{t=1}^{r}\sum_{i=1}^{n}W_{ni}\left(x\right)\left(z_{i}\beta +h\left(x\right)+\sigma_{i}\varepsilon_{i}^{\left(t\right)}-z_{i}{\hat{\beta }}_{r,n}^{\left(WLS\right)}\right)-h\left(x\right)\\ & =\frac{1}{r}\sum_{t=1}^{r}\sum_{i=1}^{n}W_{ni}\left(x\right)z_{i}\left(\beta -{\hat{\beta }}_{r,n}^{\left(WLS\right)}\right)-\overset{⌣}{h}\left(x\right)+\frac{1}{r}\sum_{t=1}^{r}\sum_{i=1}^{n}W_{ni}\left(x\right)\sigma_{i}\varepsilon_{i}^{\left(t\right)}\\ & ≜J_{r,n}^{\left(1\right)}-J_{r,n}^{\left(2\right)}+J_{r,n}^{\left(3\right)}. \end{array}$$

By $C_{p}$-inequality, we derive that

(42) $$E{\left|{\tilde{h}}_{r,n}\left(x\right)-h\left(x\right)\right|}^{p}\leq 3^{p-1}\left(E{\left|J_{r,n}^{\left(1\right)}\right|}^{p}+E{\left|J_{r,n}^{\left(2\right)}\right|}^{p}+E{\left|J_{r,n}^{\left(3\right)}\right|}^{p}\right).$$

By (23) and $\left(C_{5}\right)$, we have

(43) $$\underset{\min\left(r,n\right)\to \infty }{\lim}\underset{x\in M}{\sup}E{\left|J_{r,n}^{\left(1\right)}\right|}^{p}=0.$$

From (40), it follows that

(44) $$\underset{\min\left(r,n\right)\to \infty }{\lim}\underset{x\in M}{\sup}E{\left|J_{r,n}^{\left(2\right)}\right|}^{p}=0.$$

By $\left(C_{1}\right)$ (ii) and $\left(C_{2}\right)$ (i) (ii), we can get that

$$\underset{i\geq 1,x\in M}{\sup}\left|W_{ni}\left(x\right)\sigma_{i}\right|\leq C\underset{i\geq 1,x\in M}{\sup}\left|W_{ni}\left(x\right)\right|=O\left(n^{-\alpha }\right),$$

and

$$\underset{x\in M}{\sup}\sum_{i=1}^{n}\left|W_{ni}\left(x\right)\sigma_{i}\right|\leq C\underset{x\in M}{\sup}\sum_{i=1}^{n}\left|W_{ni}\left(x\right)\right|=O\left(1\right).$$

Hence, by (i) of Lemma 4, we have

(45) $$\underset{\min\left(r,n\right)\to \infty }{\lim}\underset{x\in M}{\sup}E{\left|J_{r,n}^{\left(3\right)}\right|}^{p}=0.$$

Therefore, (25) follows from (42)–(45).

The proof of (24) is similar to that of (25), so we omit the details here.

This completes the proof of Theorem 1. ☐

**Proof of Theorem** **2.** We first prove (26). By (31), we can get that

$$\begin{array}{cc} {\hat{\beta }}_{r,n}^{\left(LS\right)}-\beta & ={\tilde{T}}_{n}^{-2}\left[\frac{1}{r}\sum_{t=1}^{r}\sum_{i=1}^{n}\sigma_{i}{\tilde{z}}_{i}\varepsilon_{i}^{\left(t\right)}-\sum_{j=1}^{n}{\tilde{z}}_{j}\left(\frac{1}{r}\sum_{t=1}^{r}\sum_{i=1}^{n}W_{ni}\left(x_{j}\right)\sigma_{i}\varepsilon_{i}^{\left(\left.t\right)\right.}\right)+\sum_{i=1}^{n}{\tilde{z}}_{i}\overset{⌣}{h}(x_{i})\right]\\ & ≜K_{r,n}^{\left(1\right)}-K_{r,n}^{\left(2\right)}+K_{r,n}^{\left(3\right)}. \end{array}$$

Hence, it follows by $C_{p}$-inequality that

(46) $$E{\left|n^{\frac{1}{4}}\left({\hat{\beta }}_{r,n}^{\left(LS\right)}-\beta \right)\right|}^{p}\;\leq 3^{p-1}\left(E{\left|n^{\frac{1}{4}}K_{r,n}^{\left(1\right)}\right|}^{p}+E{\left|n^{\frac{1}{4}}K_{r,n}^{\left(2\right)}\right|}^{p}+E{\left|n^{\frac{1}{4}}K_{r,n}^{\left(3\right)}\right|}^{p}\right).$$

Observe that $K_{r,n}^{\left(1\right)}=\frac{1}{r}\sum_{t=1}^{r}\sum_{i=1}^{n}\left(T_{n}^{-2}\sigma_{i}{\tilde{z}}_{i}\right)\varepsilon_{i}^{\left(t\right)}≜\frac{1}{r}\sum_{t=1}^{r}\sum_{i=1}^{n}b_{ni}\varepsilon_{i}^{\left(t\right)}$. Hence, it follows from $\left(C_{1}\right)$ (i) (ii) and (13) that

$$\underset{1\leq i\leq n}{\max}\left|b_{ni}\right|\leq C\underset{1\leq i\leq n}{\max}\left|{\tilde{z}}_{i}\right|{\tilde{T}}_{n}^{-1}\cdot {\tilde{T}}_{n}^{-1}=O\left(n^{-1/2}\right)$$

and

$$\sum_{i=1}^{n}\left|b_{ni}\right|\leq C\sum_{i=1}^{n}\left|{\tilde{z}}_{i}\right|{\tilde{T}}_{n}^{-2}=O\left(1\right).$$

Thus, by (ii) of Lemma 4, we have

(47) $$\underset{\min\left(r,n\right)\to \infty }{\lim}E{\left|n^{\frac{1}{4}}K_{r,n}^{\left(1\right)}\right|}^{p}=0.$$

Note that $I_{r,n}^{\left(2\right)}=\frac{1}{r}\sum_{t=1}^{r}\sum_{i=1}^{n}\left(\sum_{j=1}^{n}{\tilde{T}}_{n}^{-2}\sigma_{i}{\tilde{z}}_{i}W_{ni}\left(x_{j}\right)\right)\varepsilon_{i}^{\left(t\right)}≜\frac{1}{r}\sum_{t=1}^{r}\sum_{i=1}^{n}b_{ni}^{\prime }\varepsilon_{i}^{\left(t\right)}$. Hence, it follows from $\left(C_{2}\right)$ (i) (iii) and (13) that

$$\underset{1\leq i\leq n}{\max}\left|b_{ni}^{\prime }\right|\leq C\underset{i\geq 1,x\in M}{\sup}\left|W_{ni}\left(x\right)\right|\cdot \sum_{i=1}^{n}\left|{\tilde{z}}_{i}\right|{\tilde{T}}_{n}^{-2}=O\left(n^{-\frac{1}{2}}\right)$$

and

$$\sum_{i=1}^{n}\left|b_{ni}^{\prime }\right|\leq C\underset{x\in M}{\sup}\sum_{i=1}^{n}\left|W_{ni}\left(x\right)\right|\cdot \sum_{i=1}^{n}\left|{\tilde{z}}_{i}\right|{\tilde{T}}_{n}^{-2}=O\left(1\right)$$

Thus, by (ii) of Lemma 4, one can get that

(48) $$\underset{\min\left(r,n\right)\to \infty }{\lim}E{\left|n^{\frac{1}{4}}K_{r,n}^{\left(2\right)}\right|}^{p}=0.$$

By $\left(C_{1}\right)$ (iv), $\left(C_{2}\right)$ (i) (iii), and $\left(C_{4}\right)$, and similarly to the proof of (40), we can get that

(49) $$\underset{x\in M}{\sup}\left|\overset{⌣}{h}\left(x\right)\right|=o(n^{-\frac{1}{4}}).$$

Hence,

$$\left|n^{\frac{1}{4}}K_{r,n}^{\left(3\right)}\right|\leq n^{\frac{1}{4}}\underset{x\in M}{\sup}\left|\overset{⌣}{h}\left(x\right)\right|\cdot \sum_{i=1}^{n}\left|{\tilde{z}}_{i}\right|/{\tilde{T}}_{n}^{2}=o\left(1\right).$$

Thus,

(50) $$\underset{\min\left(r,n\right)\to \infty }{\lim}E{\left|n^{\frac{1}{4}}K_{r,n}^{\left(3\right)}\right|}^{p}=0.$$

Therefore, (26) follows from (46)–(48) and (50).

Next, we will prove (27). In view of (11), we have

$$\begin{array}{cc} {\hat{h}}_{r,n}\left(x\right)-h\left(x\right) & =\frac{1}{r}\sum_{t=1}^{r}\sum_{i=1}^{n}W_{ni}\left(x\right)\left(z_{i}\beta +h\left(x\right)+\sigma_{i}\varepsilon_{i}^{\left(t\right)}-z_{i}{\hat{\beta }}_{r,n}^{\left(LS\right)}\right)-h\left(x\right)\\ & =\frac{1}{r}\sum_{t=1}^{r}\sum_{i=1}^{n}W_{ni}\left(x\right)z_{i}\left(\beta -{\hat{\beta }}_{r,n}^{\left(LS\right)}\right)-\overset{⌣}{h}\left(x\right)+\frac{1}{r}\sum_{t=1}^{r}\sum_{i=1}^{n}W_{ni}\left(x\right)\sigma_{i}\varepsilon_{i}^{\left(t\right)}\\ & ≜L_{r,n}^{\left(1\right)}-L_{r,n}^{\left(2\right)}+L_{r,n}^{\left(3\right)}. \end{array}$$

By $C_{p}$-inequality, we derive that

(51) $$E{\left|n^{\frac{1}{4}}\left({\hat{h}}_{r,n}\left(x\right)-h\left(x\right)\right)\right|}^{p}\;\;\leq 3^{p-1}\left(E{\left|n^{\frac{1}{4}}L_{r,n}^{\left(1\right)}\right|}^{p}+E{\left|n^{\frac{1}{4}}L_{r,n}^{\left(2\right)}\right|}^{p}+E{\left|n^{\frac{1}{4}}L_{r,n}^{\left(3\right)}\right|}^{p}\right).$$

By (26) and $\left(C_{5}\right)$, we have

(52) $$\underset{\min\left(r,n\right)\to \infty }{\lim}\underset{x\in M}{\sup}E{\left|n^{\frac{1}{4}}L_{r,n}^{\left(1\right)}\right|}^{p}=0.$$

From (49), it follows that

(53) $$\underset{\min\left(r,n\right)\to \infty }{\lim}\underset{x\in M}{\sup}E{\left|n^{\frac{1}{4}}L_{r,n}^{\left(2\right)}\right|}^{p}=0.$$

By $\left(C_{1}\right)$ (ii) and $\left(C_{2}\right)$ (iii), we can get that

$$\underset{i\geq 1,x\in M}{\sup}\left|W_{ni}\left(x\right)\sigma_{i}\right|\leq C\underset{i\geq 1,x\in M}{\sup}\left|W_{ni}\left(x\right)\right|=O\left(n^{-\frac{1}{2}}\right).$$

By $\left(C_{1}\right)$ (ii) and $\left(C_{2}\right)$ (i), we can get that

$$\underset{x\in M}{\sup}\sum_{i=1}^{n}\left|W_{ni}\left(x\right)\sigma_{i}\right|\leq C\underset{x\in M}{\sup}\sum_{i=1}^{n}\left|W_{ni}\left(x\right)\right|=O\left(1\right).$$

Hence, by (ii) of Lemma 3.4, we have

(54) $$\underset{\min\left(r,n\right)\to \infty }{\lim}\underset{x\in M}{\sup}E{\left|n^{\frac{1}{4}}L_{r,n}^{\left(3\right)}\right|}^{p}=0.$$

Therefore, (27) follows from (51)–(54).

The proof of (28) is similar to that of (27), so we omit the details here.

This completes the proof of Theorem 2. ☐

## 7. Proofs of Lemmas

**Proof of Lemma** **4.** We only prove (20), as the proof of (21) is analogous. Denote

$$\varepsilon_{1i}^{\left(t\right)}=-r^{\frac{1}{p}}I\left(\varepsilon_{i}^{\left(t\right)}<-r^{\frac{1}{p}}\right)+\varepsilon_{i}^{\left(t\right)}I\left(\left|\varepsilon_{i}^{\left(t\right)}\right|\leq r^{\frac{1}{p}}\right)+r^{\frac{1}{p}}I\left(\varepsilon_{i}^{\left(t\right)}>r^{\frac{1}{p}}\right),\;\;\varepsilon_{2i}^{\left(t\right)}=\varepsilon_{i}^{\left(t\right)}-\varepsilon_{1i}^{\left(t\right)}=\left(\varepsilon_{i}^{\left(t\right)}+r^{\frac{1}{p}}\right)I\left(\varepsilon_{i}^{\left(t\right)}<-r^{\frac{1}{p}}\right)+\left(\varepsilon_{i}^{\left(t\right)}-r^{\frac{1}{p}}\right)I\left(\varepsilon_{i}^{\left(t\right)}>r^{\frac{1}{p}}\right),\;\;\varepsilon_{i}^{\prime \left(t\right)}=\varepsilon_{1i}^{\left(t\right)}-E\varepsilon_{1i}^{\left(t\right)}$$

and

$$\varepsilon_{i}^{\prime \prime \left(t\right)}=\varepsilon_{2i}^{\left(t\right)}-E\varepsilon_{2i}^{\left(t\right)}.$$

Without loss of generality, we can assume that $c_{ni}\left(v\right)>0$ (otherwise, we use ${c_{ni}}^{+}\left(v\right)$ and ${c_{ni}}^{-}\left(v\right)$ instead of $c_{ni}\left(v\right)$ respectively, and note that $c_{ni}\left(v\right)={c_{ni}}^{+}\left(v\right)-{c_{ni}}^{-}\left(v\right)$). Hence, we known by Lemma 1 that $\left\{c_{ni}\left(v\right)\varepsilon_{i}^{\left(t\right)},1\leq t\leq r,1\leq i\leq n\right\}$, $\left\{c_{ni}\left(v\right)\varepsilon_{i}^{\prime \left(t\right)},1\leq t\leq r,1\leq i\leq n\right\}$, and $\left\{c_{ni}\left(v\right)\varepsilon_{i}^{\prime \prime \left(t\right)},1\leq t\leq r,\right.$ $\left.1\leq i\leq n\right\}$ are still AANA sequences with mixing coefficients $\left\{q^{\left(t\right)}\left(i\right)=q\left(\left(t-1\right)n+i\right),1\leq t\leq r,1\leq i\leq n\right\}$ and zero mean. Note that $\varepsilon_{i}^{\left(t\right)}=\varepsilon_{i}^{\prime \left(t\right)}+\varepsilon_{i}^{\prime \prime \left(t\right)}$. Hence, by the $C_{p}$-inequality, we have

(55) $$\begin{array}{cc} E{\left|\frac{1}{r}\sum_{t=1}^{r}\sum_{i=1}^{n}c_{ni}\left(v\right)\varepsilon_{i}^{\left(t\right)}\right|}^{p} & \leq C\left(E{\left|\frac{1}{r}\sum_{t=1}^{r}\sum_{i=1}^{n}c_{ni}\left(v\right)\varepsilon_{i}^{\prime \left(t\right)}\right|}^{p}+E{\left|\frac{1}{r}\sum_{t=1}^{r}\sum_{i=1}^{n}c_{ni}\left(v\right)\varepsilon_{i}^{\prime \prime \left(t\right)}\right|}^{p}\right)\\ & =C\left(G_{r,n}^{\left(1\right)}+G_{r,n}^{\left(2\right)}\right). \end{array}$$

When 1<p≤2, for every s>p, it follows from (16)–(19), $E{\left|\varepsilon \right|}^{p}<\infty$, and Lemma 3 that

(56) $$\begin{array}{cc} \underset{v\in M}{\sup}G_{r,n}^{\left(1\right)} & \leq {\left(\underset{v\in M}{\sup}E{\left|\frac{1}{r}\sum_{t=1}^{r}\sum_{i=1}^{n}c_{ni}\left(v\right)\varepsilon_{i}^{\prime \left(t\right)}\right|}^{s}\right)}^{p/s}\\ & \leq C{\left[{\left(\frac{1}{r}\right)}^{s}\left(\underset{v\in M}{\sup}E{\left|\sum_{t=1}^{r}\sum_{i=1}^{n}c_{ni}\left(v\right)\varepsilon_{i}^{\prime \left(t\right)}\right|}^{s}\right)\right]}^{p/s}\\ & \leq C{\left(\frac{1}{r}\right)}^{p}{\left(\underset{v\in M}{\sup}\left(C_{s}\sum_{t=1}^{r}\sum_{i=1}^{n}E{\left|c_{ni}\left(v\right)\varepsilon_{i}^{\prime \left(t\right)}\right|}^{s}\right)\right)}^{p/s}\\ & \leq C{\left(\frac{1}{r}\right)}^{p}{\left(r^{s/p}n^{-\alpha \left(s-1\right)}\right)}^{p/s}\leq Cr^{1-p} \end{array}$$

and

(57) $$\begin{array}{cc} \underset{v\in M}{\sup}G_{r,n}^{\left(2\right)} & \leq C{\left(\frac{1}{r}\right)}^{p}\underset{v\in M}{\sup}E{\left|\sum_{t=1}^{r}\sum_{i=1}^{n}c_{ni}\left(v\right)\varepsilon_{i}^{\prime \prime \left(t\right)}\right|}^{p}\\ & \leq C{\left(\frac{1}{r}\right)}^{p}\underset{v\in M}{\sup}\left(C_{p}\sum_{t=1}^{r}\sum_{i=1}^{n}E{\left|c_{ni}\left(v\right)\varepsilon_{i}^{\prime \prime \left(t\right)}\right|}^{p}\right)\\ & \leq C{\left(\frac{1}{r}\right)}^{p}rn^{-\alpha \left(p-1\right)}\leq Cr^{1-p}. \end{array}$$

Therefore, (20) follows from (55)–(57) for 1<p≤2.

When p>2, for every s>p, it follows from (15), (17)–(19), $E{\left|\varepsilon \right|}^{p}<\infty$, and Lemma 3 that

(58) $$\begin{array}{cc} \underset{v\in M}{\sup}G_{r,n}^{\left(1\right)} & \leq {\left(\underset{v\in M}{\sup}E{\left|\frac{1}{r}\sum_{t=1}^{r}\sum_{i=1}^{n}c_{ni}\left(v\right)\varepsilon_{i}^{\prime \left(t\right)}\right|}^{s}\right)}^{p/s}\\ & \leq C{\left[{\left(\frac{1}{r}\right)}^{s}\left(\underset{v\in M}{\sup}E{\left|\sum_{t=1}^{r}\sum_{i=1}^{n}c_{ni}\left(v\right)\varepsilon_{i}^{\prime \left(t\right)}\right|}^{s}\right)\right]}^{p/s}\\ & \leq C{\left(\frac{1}{r}\right)}^{p}\left(\underset{v\in M}{\sup}\left(C_{s}\left({\left[1+C\sum_{t=1}^{\infty }\sum_{i=1}^{\infty }{\left(q^{\left(t\right)}\left(i\right)\right)}^{\tilde{u}}\right]}^{s-1}\right.\sum_{t=1}^{r}\sum_{i=1}^{n}E{\left|c_{ni}\left(v\right)\varepsilon_{i}^{\prime \left(t\right)}\right|}^{s}\right.\right.\\ & \;{\left.\left.\left.+{\left[\sum_{t=1}^{r}\sum_{i=1}^{n}E{\left(c_{ni}\left(v\right)\varepsilon_{i}^{\prime \left(t\right)}\right)}^{2}\right]}^{s/2}\right)\right)\right)}^{p/s}\\ & \leq C{\left(\frac{1}{r}\right)}^{p}\left(\underset{v\in M}{\sup}\sum_{t=1}^{r}\sum_{i=1}^{n}E{\left|c_{ni}\left(v\right)\varepsilon_{i}^{\prime \left(t\right)}\right|}^{s}\right.\\ & \;{\left.+\underset{v\in M}{\sup}{\left[\sum_{t=1}^{r}\sum_{i=1}^{n}E{\left(c_{ni}\left(v\right)\varepsilon_{i}^{\prime \left(t\right)}\right)}^{2}\right]}^{s/2}\right)}^{p/s}\\ & \leq C{\left(\frac{1}{r}\right)}^{p}{\left(r^{s/p}n^{-\alpha \left(s-1\right)}+r^{s/2}n^{-\alpha s/2}\right)}^{p/s}\\ & \leq Cr^{-p/2} \end{array}$$

and

(59) $$\begin{array}{cc} \underset{v\in M}{\sup}G_{r,n}^{\left(2\right)} & \leq C{\left(\frac{1}{r}\right)}^{p}\left(\underset{v\in M}{\sup}E{\left|\sum_{t=1}^{r}\sum_{i=1}^{n}c_{ni}\left(v\right)\varepsilon_{i}^{\prime \prime \left(t\right)}\right|}^{p}\right.\\ & \leq C{\left(\frac{1}{r}\right)}^{p}\underset{v\in M}{\sup}\left(C_{p}\left({\left[1+C\sum_{t=1}^{\infty }\sum_{i=1}^{\infty }{\left(q^{\left(t\right)}\left(i\right)\right)}^{\tilde{u}}\right]}^{p-1}\sum_{t=1}^{r}\sum_{i=1}^{n}E{\left|c_{ni}\left(v\right)\varepsilon_{i}^{\prime \prime \left(t\right)}\right|}^{p}\right.\right.\\ & \;\left.\left.+{\left[\sum_{t=1}^{r}\sum_{i=1}^{n}E{\left(c_{ni}\left(v\right)\varepsilon_{i}^{\prime \left(t\right)}\right)}^{2}\right]}^{p/2}\right)\right)\\ & \leq C{\left(\frac{1}{r}\right)}^{p}\left(rn^{-\alpha \left(p-1\right)}+r^{p/2}n^{-\alpha p/2}\right)\\ & \leq Cr^{-p/2}. \end{array}$$

Therefore, (20) follows from (55), (58) and (59) for p>2.

This completes the proof of Lemma 4. ☐

## Figures and Tables

**Table 1:** The MSEs of ${\hat{\beta }}_{r,n}^{\left(LS\right)}$ with β=3.5 and $h\left(x\right)=\cos\left(\pi x\right)$.

x	*n* = 500	*n* = 1000	*n* = 1500	*n* = 2000
0.3	0.0115	0.0054	0.0036	0.0028
0.6	0.0112	0.0053	0.0041	0.0029
0.9	0.0113	0.0052	0.0038	0.0026

**Table 2:** The MSEs of ${\hat{h}}_{r,n}\left(x\right)$ with β=3.5 and $h\left(x\right)=\cos\left(\pi x\right)$.

x	*n* = 500	*n* = 1000	*n* = 1500	*n* = 2000
0.3	0.0174	0.0099	0.0077	0.0053
0.6	0.0182	0.0100	0.0071	0.0059
0.9	0.0195	0.0093	0.0073	0.0060

## Data Availability

The original contributions presented in this study are included in the article. Further inquiries can be directed to the corresponding author.
